# Improved Performance of NbO_x_ Resistive Switching Memory by In-Situ N Doping

**DOI:** 10.3390/nano12061029

**Published:** 2022-03-21

**Authors:** Jing Xu, Yuanyuan Zhu, Yong Liu, Hongjun Wang, Zhaorui Zou, Hongyu Ma, Xianke Wu, Rui Xiong

**Affiliations:** 1 School of Physics and Technology, and the Key Laboratory of Artificial Micro/Nano Structures of Ministry of Education, Wuhan University, Wuhan 430072, China; jxu_materials@whu.edu.cn (J.X.); zrzou@whu.edu.cn (Z.Z.); mahongyu@whu.edu.cn (H.M.); xiankewu@whu.edu.cn (X.W.); 2 Department of Physics, Shaanxi University of Science and Technology, Xi’an 710021, China; zhuyuanyuan@sust.edu.cn (Y.Z.); wanghongjun@sust.edu.cn (H.W.)

**Keywords:** NbO_x_, *N*-doping, memory device, resistance switching, oxygen vacancy

## Abstract

Valence change memory (VCM) attracts numerous attention in memory applications, due to its high stability and low energy consumption. However, owing to the low on/off ratio of VCM, increasing the difficulty of information identification hinders the development of memory applications. We prepared N-doped NbO_x_:N films (thickness = approximately 15 nm) by pulsed laser deposition at 200 °C. N-doping significantly improved the on/off ratio, retention time, and stability of the Pt/NbO_x_:N/Pt devices, thus improving the stability of data storage. The Pt/NbO_x_:N/Pt devices also achieved lower and centralized switching voltage distribution. The improved performance was mainly attributed to the formation of oxygen vacancy (V_O_) + 2N clusters, which greatly reduced the ionic conductivity and total energy of the system, thus increasing the on/off ratio and stability. Moreover, because of the presence of Vo + 2N clusters, the conductive filaments grew in more localized directions, which led to a concentrated distribution of SET and RESET voltages. Thus, in situ *N*-doping is a novel and effective approach to optimize device performances for better information storage and logic circuit applications.

## 1. Introduction

Resistive random access memory (RRAM) is highly popular, owing to its high write and erase speeds, high storage density, and multi-level storage; additionally, it is considered the most potential candidate for next-generation memory [1,2,3,4,5,6,7]. Several studies have reported the excellent performance of RRAM, such as MoS_2_ filament transistors with high on/off ratio (2.6 × 10^9^), power-efficient h-BN memories, and cellulose memories with a significant on/off ratio (10^6^) and low SET/RESET voltages (<0.5 V) [8,9,10]. However, the cross array structure of contemporary resistive memory devices has a serious crosstalk current problem. NbO_x_-based devices that exhibit either threshold or memory resistance switching (RS), according to the content of oxygen vacancies in the NbO_x_ film [11], can self-assemble as 1Selector-1RRAM (1S1R) devices to suppress crosstalk current. Several studies have reported the threshold switching characteristics of NbO_x_ devices [12,13,14], but few studies have investigated the optimization of NbO_x_ memory RS characteristics [15,16]. The performance of NbO_x_ memristor devices is insufficient for applications; thus, it is essential to optimize the performances of NbO_x_ memristor devices. Research on optimizing memory RS characteristics of NbO_x_ can provide insights for applications of NbO_x_-based 1S1R devices.

Doping, especially N-doping, in RS functional layers has been widely used as an effective method to improve RRAM performance. Park et al. [17] demonstrated that N-doping in the HfO_x_:N layers significantly restrained the randomness distribution of Ag conductive filaments (CFs) and decreased the power consumption and AC response time of electrochemical change memory (ECM)-type Ag/HfO_x_:N/Pt/Ti devices. Sedghi et al. [18] reported that *N*-doping in Pt/Ti/Ta_2_O_5_:N/Pt effectively reduced the defect densities induced by oxygen vacancies and achieved multi-level switching. Yang et al. [19] reported that the formation of a hafnium-nitrogen complex near oxygen vacancies in a N-doped HfO_2_ system, as determined by ab initio calculations, greatly reduced the total energy of the system and increased its stability. Several studies have reported the benefits of N-doping on ECM-type RRAM [17,18,20], mainly focusing on metal CFs. However, few studies have investigated the effect of *N*-doping on VCM-type, NbO_x_-based RRAM [18], that is, on CFs with oxygen vacancy. The low on/off ratio in VCM-type, NbO_x_-based RRAM devices limits their applications in memory and logic circuits. In this paper, the purpose of N doping is twofold: to increase the resistivity of the film and improve the on/off ratio of the device by replacing O with N; in addition, in situ N-doping can promote the uniform doping of N and uniform substitution of N for O in thin films. Research on the effect of in situ N-doping on VCM-type, NbO_x_-based RRAM devices can provide insights for their applications.

In situ N-doping can ensure uniform N-doping on thin films and does not require a subsequent annealing, simplifying the preparation process and reducing excess energy consumption. In this study, chemically inert Pt electrodes were used as the top and bottom electrodes, and the influence of in situ N-doping on VCM-type, NbO_x_ RRAM devices was investigated. N was introduced via a nitrogen–oxygen mixture atmosphere during film deposition. Compared with Pt/NbO_x_/Pt device, the Pt/NbO_x_:N/Pt device had an improved on/off ratio, was more stable, and had longer retention time. Further, differences in the RS behaviors and conduction mechanisms of Pt/NbO_x_/Pt and Pt/NbO_x_:N/Pt memory devices were elucidated.

## 2. Experimental Section

Except the different deposition atmosphere on the Si/SiO_2_/Ti/Pt substrate at 200 °C, NbO_x_ and NbO_x_:N RS films were deposited by pulsed laser deposition (KrF pulsed excimer laser, COMPEX PRO 201F, Coherent Gmbh, Dieburg, Germany, wavelength = 248 nm), under the same deposition conditions (laser energy = 400 mJ, repetition frequency = 6 Hz, and bombardments with = 4000 counts). The NbO_x_ film was deposited under a pure O_2_ atmosphere, and the NbO_x_:N film was deposited under a mixed atmosphere of O_2_–N_2_ (50:8, sccm). The background pressure of the chamber was 8 × 10^−4^ Pa, and it was maintained at 20 Pa during deposition. A Nb metallic target was used as the Nb source, and the distance between the Nb target and substrate was 4 cm. A circular pore Pt (thickness = 100 nm, diameter = 300 μm) was used as the top electrode.

The microstructure information of the prepared films was characterized using X-Ray diffraction (XRD, Rigaku SmartLab, Rigaku Corporation, Tokyo, Japan). The prepared films were cut using focused ion beam (FIB, GAIA3 XMH, TESCAN, Brno, Czech Republic). The film thickness and elemental composition were determined using transmission electron microscopy (TEM, JEM-F200, JEOL, Tokyo, Japan) with energy dispersive X-Ray spectroscopy (EDX, JEM-F200, JEOL, Tokyo, Japan). Surface morphologies and element mapping of the prepared films were characterized using scanning electron microscopy (SEM, Zeiss SIGMA, Carl Zeiss Microscopy Ltd., Cambridge, UK) with energy disperse spectroscopy (EDS, Aztec Energy, Oxford Instruments Nanoanalysis, Oxford, UK). The valence states of the elements and Fermi levels of the prepared films were measured using X-Ray photoelectron spectroscopy (XPS, ESCALAB 250Xi, Thermo Fisher Scientific, Waltham, MA, USA) and ultraviolet photoelectron spectroscopy (UPS, ESCALAB 250Xi, Thermo Fisher Scientific, Waltham, MA, USA). Electrical properties were measured using a semiconductor parameter analyzer (Keithley 4200-SCS, Keithley Instruments, Inc., Cleveland, OH, USA), with the DC mode at room temperature under an air atmosphere.

## 3. Results and Discussion

**Figure 1:**
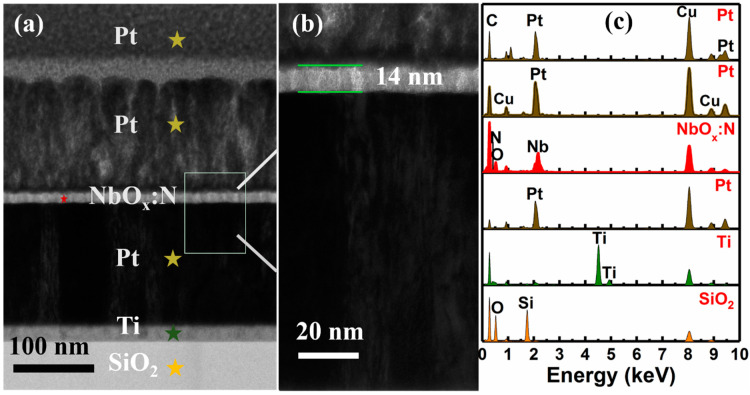
(**a**) Cross-sectional TEM of Si/SiO_2_/Ti/Pt/NbO_x_:N multi-film; (**b**) enlarged view of the NbO_x_:N film in a square area of (**a**); (**c**) EDX point scanning of each layer in a pentagram region in (**a**).

A multilayer substrate, Si/SiO_2_ (300 nm)/Ti (20 nm)/Pt (150 nm), was used in this study. To determine the thickness of the prepared film, TEM was used to intuitively observe the thickness of each layer of the NbO_x_:N film. The thickness of each layer of the NbO_x_:N film is shown in Figure 1a. High-resolution TEM was conducted on a square area of the NbO_x_:N film of Figure 1a; as shown in Figure 1b, the thickness of NbO_x_:N film was 14 nm. Due to the similar preparation conditions of the NbO_x_ and NbO_x_:N films, it can be inferred that the thickness of the NbO_x_ film was also about approximately 15 nm. Figure 1c shows the EDX point scanning results of the corresponding layers in a pentagram region in Figure 1a. In the EDX scanning results of the NbO_x_:N layer, peaks corresponding to Nb, N, and O are observed; the appearance of an N peak indicates successful N-doping on the NbO_x_ film. The observed film was placed on a Cu network with a C film, because of which, the peaks of Cu and C also appeared in the EDX scanning results.

**Figure 2:**
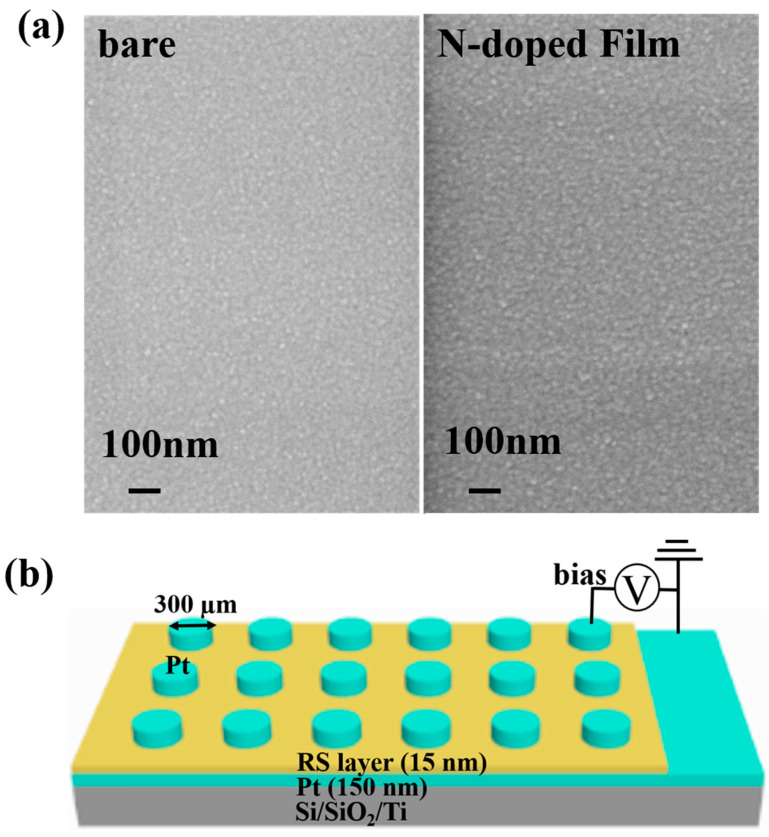
(**a**) Surface morphologies of bare NbO_x_ and N-doped films. (**b**) Schematic diagram of the Pt/RS layer/Pt memory device (RS layer: NbO_x_ or NbO_x_:N).

The effect of N-doping on the surface morphologies of NbO_x_ films was observed using SEM profiles. Figure 2a shows that bare as well as N-doped films were continuous and dense, and the particles forming the films were closely and neatly packed. A comparison of the two films shows that the surface of the NbO_x_:N film was more dense, size of the particles forming the film was more uniform, and particles were more closely arranged. Denser film means fewer defects in the NbO_x_:N film, which may be related to the passivation of oxygen vacancies by N doping. Fewer oxygen vacancies in NbO_x_:N film may result in an abrupt switching behavior in the RS device [16]. Due to the deposition temperate of 200 °C, which is lower than the crystallization temperature of the NbO_x_ film, apart from the substrate peak, there was no crystalline peak of NbO_x_ in the XRD results, as shown in Figure S1. The metal–insulator–metal structure schematic of the Si/SiO_2_/Ti/Pt/RS layer/Pt device is shown in Figure 2b. The top and bottom electrodes of the device were Pt electrodes; at the top electrode, a voltage was supplied, and the bottom electrode was grounded.

**Figure 3:**
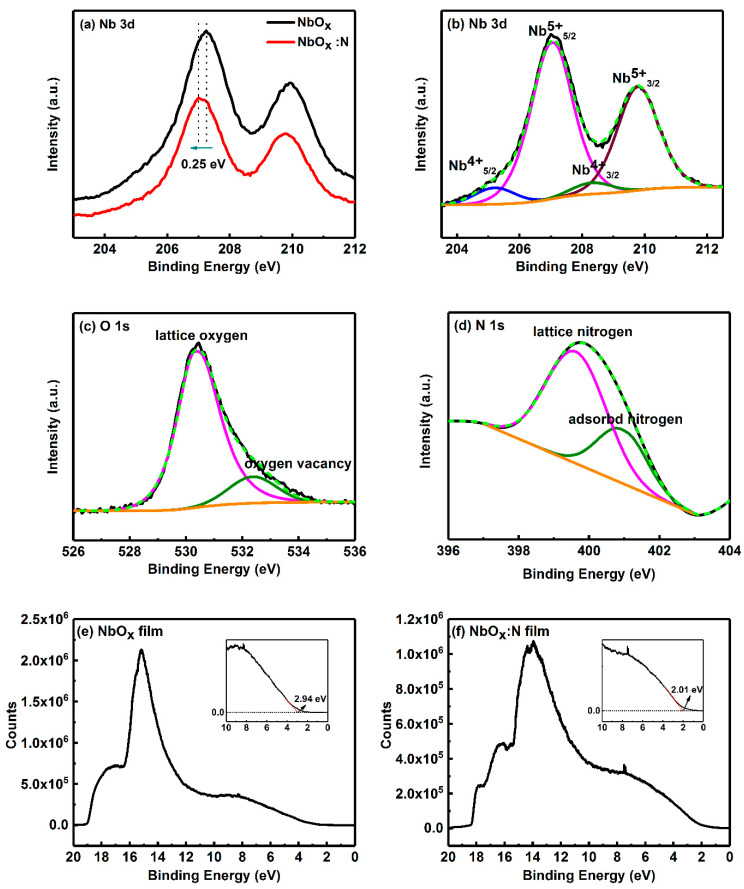
(**a**) Comparison of Nb 3d core level spectra in NbO_x_ and NbO_x_:N films. (**b**) Nb 3d, (**c**) O 1s, and (**d**) N 1s core-level fitting spectra of NbO_x_:N film. UPS spectra of (**e**) NbO_x_ and (**f**) NbO_x_:N film, measured with −6 V bias. The red dotted line represents the Fermi edge of each sample in subfigures (**e**,**f**).

XPS profiles were used to confirm the successful doping of nitrogen into the lattice of NbO_x_ film. The peak position of the binding energy for each element was calibrated by C 1s at 284.8 eV. The N 1s spectral lines were fitted after smoothing by Gaussian function. Figure 3a shows that the binding energy of Nb 3d shifted toward a lower binding energy of 0.25 eV after N incorporation, which is mainly because N atoms are less electronegative than O atoms. The result implied the presence of the Nb–O–N group, which is agreement with the findings of another similar report [17]. Figure 3b shows that two strong peaks at binding energy of 207.05 and 209.79 eV, implying that the chemical environment around Nb was the same as Nb_2_O_5_ [21]_._ Two weak peaks appear at binding energy of 205.19 and 208.30 eV, which correspond to spin orbital splits of 3d_5/2_ and 3d_3/2_ of Nb^4+^, respectively. Figure 3c shows two peaks at binding energy of 530.36 and 532.38 eV, which correspond to lattice oxygen in most oxides and oxygen vacancies, respectively [22]. The existence of oxygen vacancies suggested that the RS mechanism was related to the oxygen vacancy CF in the Pt/NbO_x_:N/Pt memory device. Figure 3d shows two peaks at binding energies of 399.54 and 400.84 eV. Higher binding energies are typically ascribed to adsorbed N_2_ in air, and a peak at binding energy of approximately 399.00 eV is ascribed to the Nb–O–N lattice that was formed by N atoms substituting oxygen atoms, which is in agreement with the results shown in Figure 2a [23,24,25]. XPS results suggested that N atoms were successfully incorporated and replaced the lattice O atoms in the NbO_x_ film. The XPS spectra of the Nb 3d and O1s core-levels of NbO_x_ films are shown in Figure S2. The UPS results of NbO_x_ and NbO_x_:N film are shown in Figure 3e,f. The Fermi level of Au was defined as Fermi zero; thus, the Fermi level of NbO_x_ film and NbO_x_:N film was 2.94 and 2.01 eV, respectively. The UPS results showed that the N-doping shifted downward the Fermi level of the NbO_x_:N film, resulting in the NbO_x_:N film with poor conductivity [26] and high resistance.

**Figure 4:**
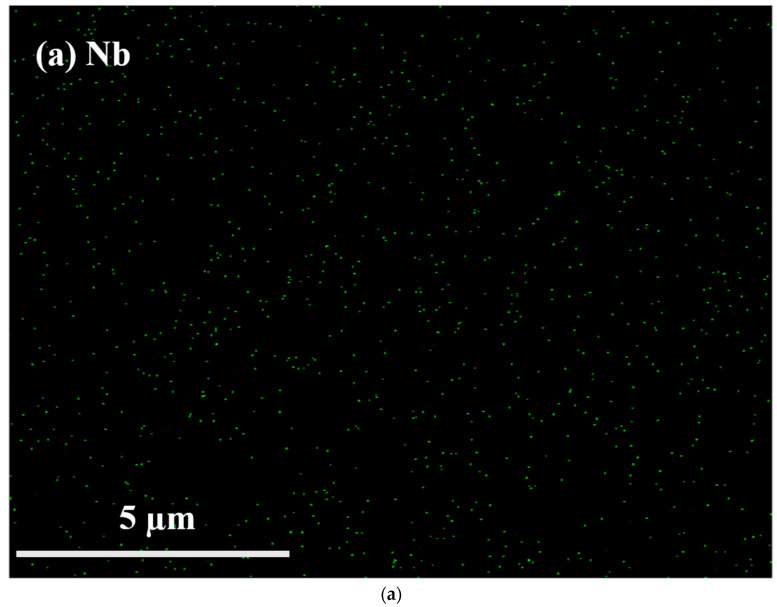

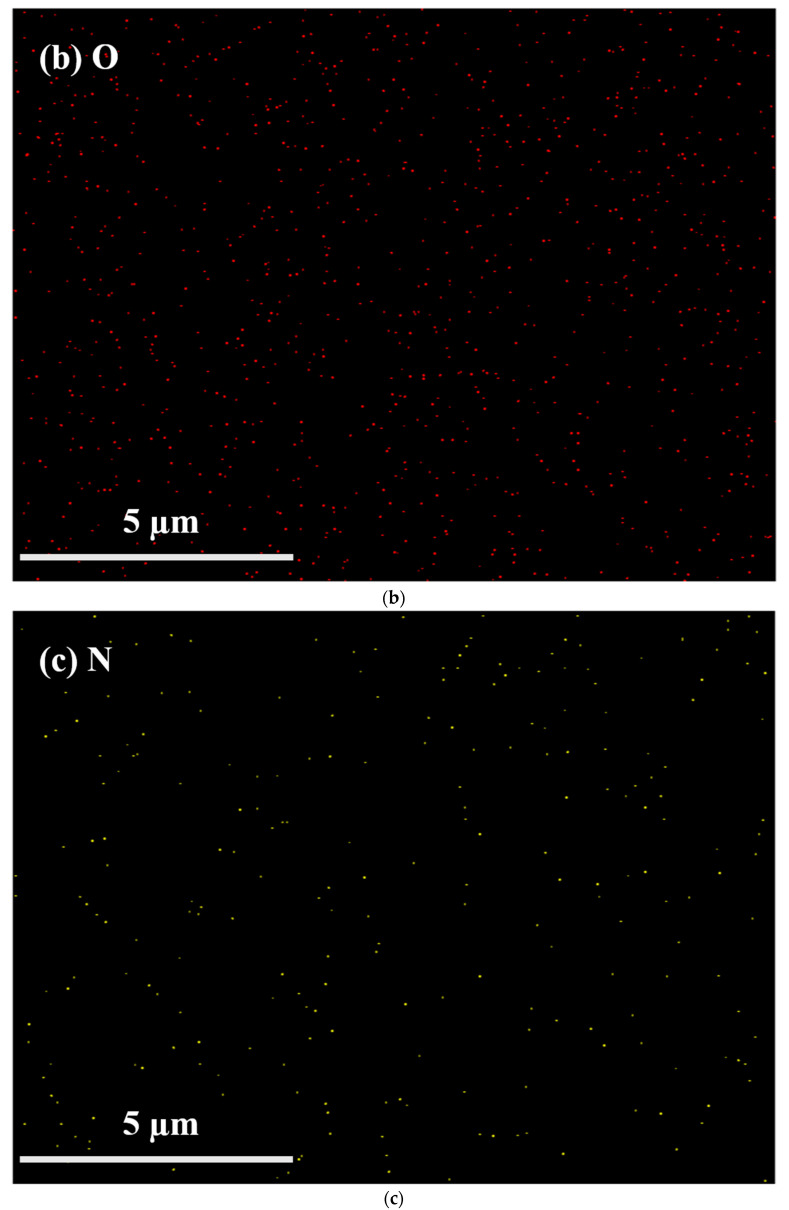
EDS mapping of (**a**) Nb, (**b**) O, and (**c**) N in NbO_x_:N films.

EDS mapping of NbO_x_:N films was used to verify uniform N-doping in the NbO_x_ films. As shown in Figure 4a,b, large quantities of Nb and O are distributed evenly and densely on the substrate. Figure 4c shows that N content was relatively small, but uniformly distributed on the substrate, indicating uniform N-doping on the NbO_x_ films.

**Figure 5:**
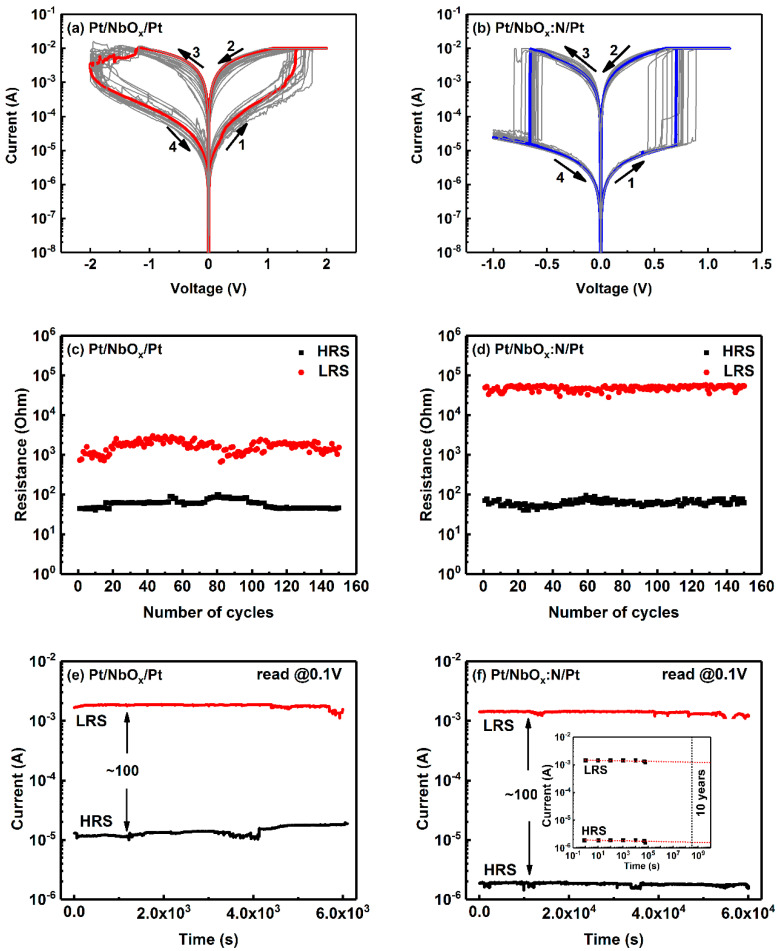
(**a**,**b**) Semi-logarithm *I-V* curves with sweeping voltages between 1.5 V and −1.5 V; (**c**,**d**) resistance distribution with the number of cycles, retention time of low-resistance state (LRS) and high-resistance state (HRS) at a voltage of 0.1 V for the (**e**) Pt/NbO_x_/Pt and (**f**) Pt/NbO_x_:N/Pt memory devices, respectively. The inset graph in (**f**) is the linear extrapolation fitting of retention time for the Pt/NbO_x_:N/Pt memory device.

Electrical properties were measured with a 4200-SCS semiconductor parameter analyzer under an air atmosphere at room temperature. The sweeping voltage of the current–voltage (*I*-*V*) curves were in the order of 0 V → 2.5 V → 0 → −2.0 V→ 0 V. *I*-*V* curves were plotted once every 5 cycles, and 20 cycles curve were plotted in total. The SET process is defined as a resistance state, switching from an HRS to a LRS, while the RESET process is defined as the opposite—that is, a resistance state switching from a LRS to an HRS. Both Pt/NbO_x_/Pt (named as M1) and Pt/NbO_x_:N/Pt (named as M2) memory devices showed bipolar RS characteristics (Figure 5a,b). Figure 5a shows that M1 exhibited a gradual RS process. Its resistance decreased, and then the reset process started at −1.1 V; however, the whole reset process was not complete until the negative sweeping voltage reached −2.0 V. The SET process for M1 occurred at approximately 1.7 V, and it was accompanied by an increase in the current, from 0.002 to 0.01 A. For M2, the SET process occurred abruptly at approximately 0.8 V, and it was accompanied by an increase in the current from 1.17 × 10^−5^ to 0.01 A. When the negative sweeping voltage reached −0.6 V, the same steep and abrupt RESET process occurred. Compared with M1, that is, without N-doping, the performance of M2, that is, with N-doping, was obviously improved. The SET and RESET processes of M2 started abruptly and instantaneously, and the on/off ratio was obviously larger than that of M1; moreover, the *I*-*V* cycle curves showed symmetrical distributions.

The detailed distribution of resistance values of M1 and M2 in HRS and LRS, along with the number of cycles, are shown in Figure 5c,d. The initial resistance of M1 was approximately 50 and 700–2000 Ω in LRS and HRS, respectively; HRS and LRS both slightly fluctuated during *I*-*V* cycle measurements. The initial resistance of M2 was approximately 70 and 30,000 Ω in LRS and HRS, respectively. The resistance values of HRS and LRS remained nearly constant during the *I*-*V* cycle measurements. The enormous difference between the resistance values of M1 and M2 in HRS implied an improvement of nearly 10 times in the on/off ratio. Due to practical limitations, namely the complexity of manual measuring, owing to the measuring time and manual effort, only approximately 150 cycles were obtained. After 150 cycles, the device could still operate normally for several cycles, without changes in the on/off ratio and switching voltage. The retention time of LRS and HRS for M1 and M2 at a voltage of 0.1 V are shown in Figure 5e,f. Figure 5e shows that the HRS and LRS M1 current values were relatively stable in the first 4000 s, and the on/off ratio was approximately 100. However, after 4000 s, the current values of HRS and LRS increased and decreased, respectively. After 6000 s, the current values of LRS began to fluctuate. However, after *N*-doping, the on/off ratio was approximately 1000, and the resistance of HRS and LRS for M2 did not show any attenuation, even after testing for 60,000 s. Using linear extrapolation, at room temperature, the retention time of the Pt/NbO_x_:N/Pt memory device was determined to be more than 10 years [27]; that is, *N*-doping significantly improved the retention time of the device.

**Figure 6:**
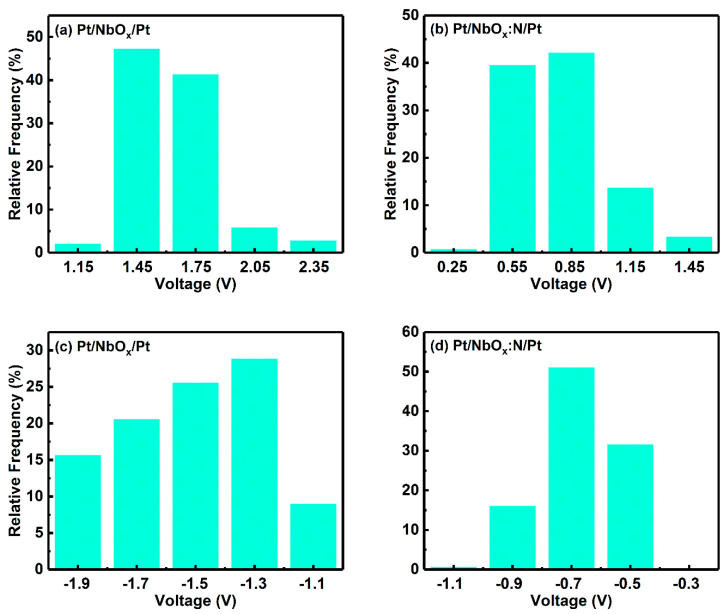
Distribution of SET voltage for (**a**) Pt/NbO_x_/Pt and (**b**) Pt/NbO_x_:N/Pt memory devices. Distribution of RESET voltage for (**c**) Pt/NbO_x_/Pt and (**d**) Pt/NbO_x_:N/Pt memory devices.

The differences in the SET and RESET voltage distributions of M1 and M2 were characterized as basis voltage distribution histograms, plotted in Figure 6. As seen in Figure 6a, the SET voltage of M1 device was distributed in the range 1.3–1.9 V, and the cumulative frequency of the two ranges was >85%; most of the SET voltage of the device was distributed in this range. As seen in Figure 6b, the SET voltage of M2 was concentrated in the range 0.4–1.3 V, cumulative frequency >85% was concentrated between 0.4 V and 1.0 V, and SET voltage was significantly lower than that of M1. As seen in Figure 6c, the RESET voltage distribution of M1 was relatively dispersed, and the voltage with a cumulative frequency of >85% was distributed between −1.2 V and −2.0 V. As seen in Figure 6d, the RESET voltage distribution of M2 was very concentrated, voltage with a cumulative frequency >85% was concentrated between −0.4 V and −0.8 V, and RESET voltage was significantly lower than that of M1. Therefore, compared with M1, the SET and RESET voltages of M2 were significantly lower, and the distribution of the RESET voltage was significantly more concentrated.

According to the above analysis of the performances of M1 and M2, M2 with N-doping exhibited better performance: increased on/off ratio, significantly improved device stability, significantly lower SET and RESET voltage, and more concentrated distribution of RESET voltage. Table 1 lists the performance parameters of NbO_x_-based memory RS devices, prepared in this paper and compared with previous reports.

**Figure 7:**
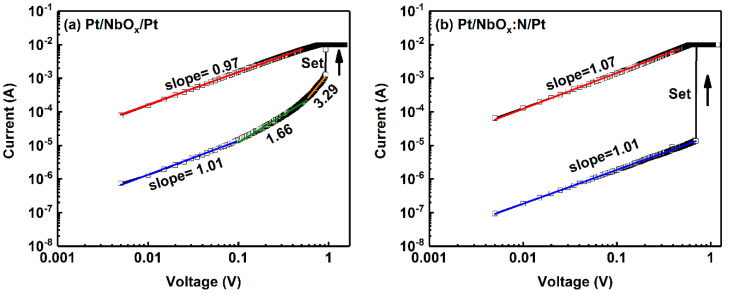
Double-logarithmic *I*-*V* curves of (**a**) Pt/NbO_x_/Pt and (**b**) Pt/NbO_x_:N/Pt memory devices. Standard errors of all fitted parameters were <0.01. Standard error values of fitting parameters and the overall fitting degree R^2^ of the equation are detailed in Tables S1 and S2.

To investigate the reason for the improved performance of the Pt/NbO_x_:N/Pt memory device, the conduction mechanisms of the Pt/NbO_x_/Pt and Pt/NbO_x_:N/Pt memory devices were analyzed. The complex nonlinear relationship between the current density and electric field can be characterized as a simple linear relationship by selecting the double-logarithmic coordinate axis; this will allow the conduction mechanism to be judged by fitting the slope of the double-logarithmic coordinate axis. Figure 7a,b shows the double-logarithmic *I*-*V* curves. Experimental data are represented by black hollow boxes, and different color fitting lines are plotted in different regions. For the Pt/NbO_x_/Pt memory device (Figure 7a), the linear fitting of the logarithmic current vs logarithmic voltage, with slope = 0.97, indicates I ∝ V^0.97^, corresponding to ohmic conductive behavior, which manifested the CF mechanism in LRS [32,33]. The fitting of HRS was divided into three different regions: I ∝ V^1.01^ in the low electric field region, I ∝ V^1.66^ in the medium electric field region, and I ∝ V^3.29^ in the high electric field region. The increasing fitting slopes of the three regions correspond to the space-charge-limited current (SCLC) mechanism in HRS [34,35]. The different conduction mechanism in LRS and HRS manifested the local Vo CF conduction mechanism in the Pt/NbO_x_/Pt memory device. The RS processes of HRS and LRS were mainly attributed to the formation and rupture of CFs.

In Figure 7b, the similar linear fitting slope of approximately 1 in LRS for the Pt/NbO_x_:N/Pt memory device indicates the same ohmic conduction mechanism in LRS. Thus, *N*-doping did not change the conduction mechanism of ohmic conduction in LRS. The linear fitting slope of HRS in different electric field regions was 1.01, which did not satisfy the theory that the slope of the SCLC mechanism increased gradually in the high electric field region. Therefore, the conduction mechanism of the Pt/NbO_x_:N/Pt device in HRS cannot be explained by the SCLC mechanism. *N*-doping passivates oxygen vacancies in oxides [36]; that is, oxygen vacancies decrease significantly after *N*-doping. The leakage current through the dielectric layer is reduced; as a result, compared to the Pt/NbO_x_/Pt device, the off-current (in the HRS) is one order of magnitude lower after N-doping (Figure 5c,d). Thus, there is improvement in the on/off ratio of the Pt/NbO_x_:N/Pt devices. The breakdown phenomenon is followed by the Ohmic region, without a Child’s law regime (Figure 7b).

In N-incorporated oxides, Vo preferably combines with two neutral N atoms to form a Vo + 2N complex [19,20,37]. In N-doped NbO_x_ films, in the presence of excess Vo, these complexes may form Vo + 2N clusters. First, principle studies have shown that the formation of clusters greatly decreases the total energy and ionic conductivity of the system and makes it more stable [17,38]; moreover, CFs in the device are more likely to grow along with clusters [39], as CFs grow in more localized directions. Therefore, in the Pt/NbO_x_:N/Pt device, the formed CFs mainly comprised of V_O_ and V_O_ + 2N clusters, which greatly decreased the ionic conductivity, resulting in the increased resistivity in the HRS of the device [18]; thus, when the LRS resistance was almost unchanged, the on/off ratio increased. The localized CFs also concentrated the distribution of SET and RESET voltages. The occurrence of Vo + 2N clusters decreased the total energy of the system and made the device more stable. Therefore, N-doping can significantly improve the performance of Pt/NbO_x_:N/Pt devices, such as the durability, stability, and on/off ratio, as well as achieve lower switching voltage and centralized voltage distribution.

**Figure 8:**
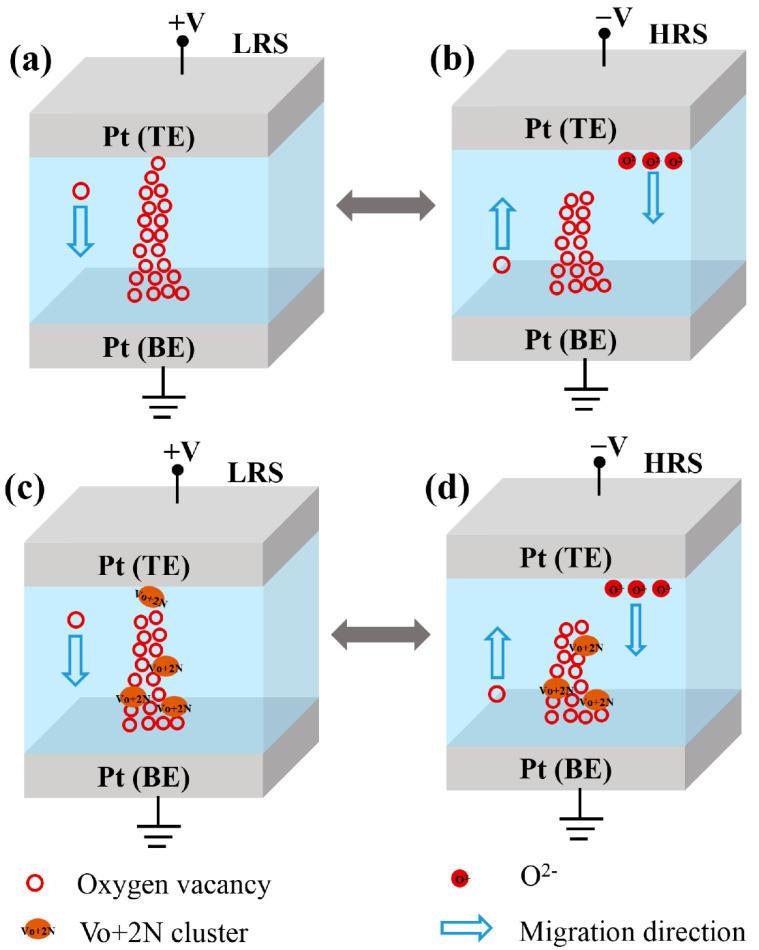
Schematic diagrams of CF formation and ruptures in LRS and HRS: (**a**,**b**) Pt/NbO_x_/Pt memory device; (**c**,**d**) Pt/NbO_x_:N/Pt memory device.

Figure 8 shows the schematic diagram of the CF formation and ruptures, both before and after N-doping. For the Pt/NbO_x_/Pt memory device, as shown in Figure 8a, after positive bias was applied to the top electrode (TE), under the influence of the electric field, the *Vo* in the NbO_x_ film moved from the TE to the bottom electrode (BE). The Vo, accumulated at the BE, developed into nuclei for the CFs, acting as extensions of the cathode, and the CFs then grew toward the anode. Finally, the BE and TE were connected via the CFs; meanwhile, the device changed from HRS to LRS. When a negative bias voltage was applied to TE, as shown in Figure 8b, most of the Joule heat accumulated in the thinnest part of the CF; at the same time, the O^2-^ moved from the TE to the BE under the influence of the electric field. The recombination of O^2-^ and Vo in the RS layer resulted in the rupture of the CFs, and the device changed to HRS [2]. For the Pt/NbO_x_:N/Pt devices, as shown in Figure 8c, when positive bias was applied to the TE, *Vo* in the NbO_x_:N film moved from the TE to the BE, under the influence of the electric field. At this time, Vo and N atoms combined to form Vo + 2N clusters. These Vo + 2N clusters gradually accumulated and extended and, eventually, formed the CFs connecting the BE and TE. When negative bias was applied to the TE, as shown in Figure 8d, O^2-^ similarly moved from the TE to the BE, under the influence of the electric field. The combination of O^2-^ and *Vo* in the RS layer led to the rupture of the CF; thus, the device returned to HRS.

## 4. Conclusions

In situ N-doping was used to prepare uniformly doped NbO_x_ films. The on/off ratio, stability and retention time of the Pt/NbO_x_:N/Pt device were better than that of the Pt/NbO_x_/Pt device; the SET and RESET voltages were significantly reduced, and the RESET voltage distribution was more concentrated. After N-doping, the formed CFs mainly comprised of V_O_ and V_O_ + 2N clusters, which greatly reduced the ionic conductivity and total energy, increasing the resistivity in the HRS of the Pt/NbO_x_:N/Pt device. Fitting the *I-V* curves of the device revealed that both devices had an ohmic conduction mechanism in LRS. In situ N-doping is an effective technique to optimize memory device performance for information storage, logic circuit, and neuromorphic computing applications.

## Figures and Tables

**Table 1:** Performance of NbO_x_-based memory RS devices, prepared in this paper and compared with previous reports.

Structure	Thickness (nm)	Deposition Method	*I-V* Characteristic	SET/RESET Voltage (V)	On/Off Ratio	Retention Time (s)	Ref
Au/Nb_2_O_5_/Pt	60	RF-sputtering	Bipolar	0.96/−1.3	10	-	[14]
Cu/Nb_2_O_5_/Pt	11–14	PLD	Bipolar	1.5/ −0.5–−1	~200	~5 × 10^3^	[16]
Pt/Nb_2_O_5_/Pt	26	E-beam evaporation	Bipolar	0.8/−0.8	20	3.6 × 10^4^	[28]
W/Nb_2_O_5_/NbO_x_/Ru	-	DC-sputtering	Bipolar	1.1/ −1.5–−1.8	~50	-	[29]
Al/Ti/SiO_2_:Nb_2_O_5_/TiN/Si	18–22	ALD	Bipolar	1/−1	<10	-	[30]
Pt/Nb_2_O_5_/Pt/TiN/SiO_2_/Si	50	DC-sputtering	Unipolar	1.7/0.8	100	1.67 × 10^4^	[31]
Pt/NbO_x_:N/Pt	~15	PLD	Bipolar	0.4–1.0/ −0.4–−0.8	~10^3^	6 × 10^4^	This work

## Data Availability

The data that support the findings of this study are available within this article and its Supplementary Materials.

## Supplementary Materials

The following supporting information can be downloaded at: https://www.mdpi.com/article/10.3390/nano12061029/s1. Figure S1: XRD profiles of NbO_x_ and NbO_x_:N films; Figure S2: (a) Nb 3d and (b) O1s core-level fitting spectra of NbO_x_ films; Table S1: Standard error values of fitting parameters and the fitting degree R^2^ of the equation in Figure 6a; Table S2: Standard error values of fitting parameters and the fitting degree R^2^ of the equation in Figure 6b.
