# Supplementary material for: Improved Performance of NbOx Resistive Switching Memory by In-Situ N Doping
Source: Nanomaterials (Basel). 2022 Mar 21;12(6):1029. doi: 10.3390/nano12061029 (PMC8949618; doi:10.3390/nano12061029)
Supplement: Supplementary file 1 [file nanomaterials-12-01029-s001.zip › nanomaterials-1597768-supplementary.pdf]

Supplementary Materials

Improved Performance of NbO_x Resistive Switching Memory by In-Situ N Doping

Jing Xu [1], Yuanyuan Zhu [2], Yong Liu [1,*], Hongjun Wang [2], Zhaorui Zou [1], Hongyu Ma [1], Xianke Wu [1] and Rui Xiong [1,*]

[1] School of Physics and Technology, and the Key Laboratory of Artificial Micro/Nano Structures of Ministry of Education, Wuhan University, Wuhan 430072, China; jxu_materials@whu.edu.cn (J.X.); zrzou@whu.edu.cn (Z.Z.); mahongyu@whu.edu.cn (H.M.); xiankewu@whu.edu.cn (X.W.)

[2] Department of Physics, Shanxi University of Science and Technology, Xi'an 710021, China; zhuyuanyuan@sust.edu.cn (Y.Z.); wanghongjun@sust.edu.cn (H.W.)

* Correspondence: yongliu@whu.edu.cn (Y.L.); xiongrui@whu.edu.cn (R.X.)

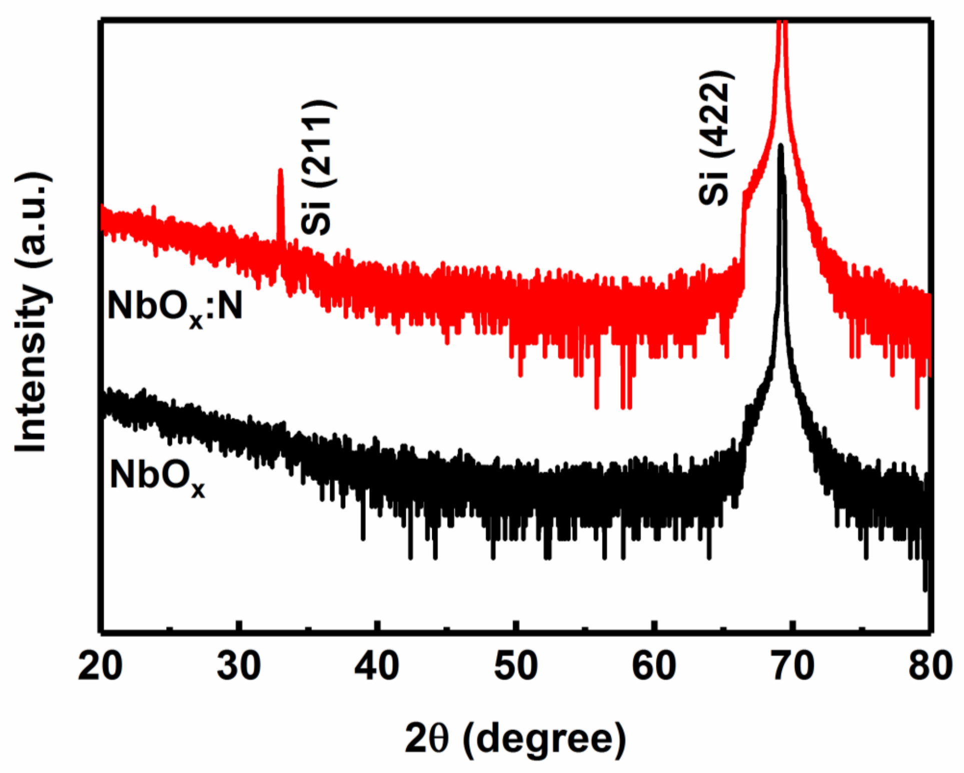

Figure S1. XRD profiles of NbO_x and NbO_x:N film.

Due to the deposition temperate of 200 °C, which is lower than the crystallization temperature of the NbO_x film, apart from the substrate peak, there was no crystalline peak of NbO_x in the XRD results, as shown in Figure S1.

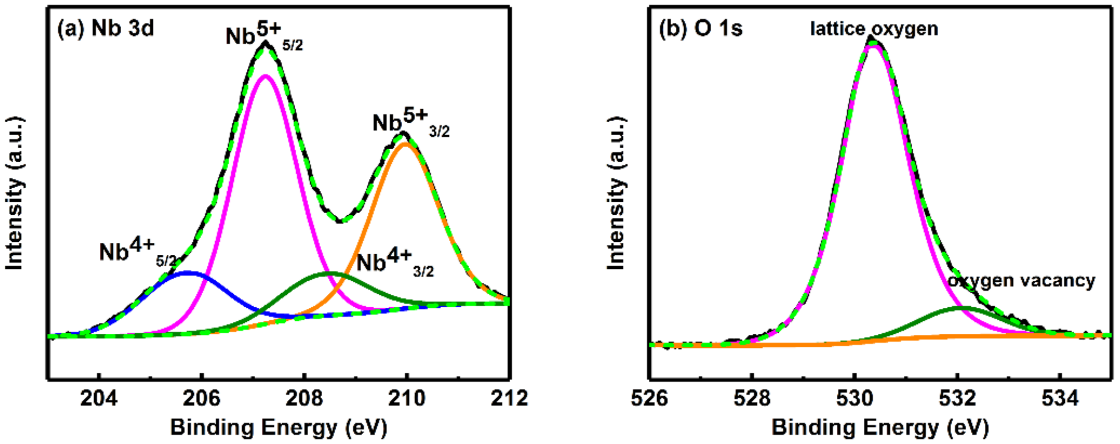

Figure S2. (**a**) Nb 3d and (**b**) O1s core-level fitting spectra of NbO_x film.

Table S1. Standard error values of fitting parameters and the fitting degree R^2 of the equation in Figure 6a.

	Intercept		Slope		Statistics
	Value	Standard Error	Value	Standard Error	Adj. R-Square
a. LRS	−1.84704	0.00169	0.96813	0.00207	0.99954
b. HRS low electric field region	−3.84937	0.0184	1.01105	0.01275	0.99714
c. HRS medium electric field region	−3.25003	0.00911	1.6635	0.01632	0.99103
d. HRS High electric field region	−2.82761	0.0048	3.28589	0.03488	0.99262

Table S2. Standard error values of fitting parameters and the fitting degree R^2 of the equation in Figure 6b.

	Intercept		Slope		Statistics
	Value	Standard Error	Value	Standard Error	Adj. R-Square
a. LRS	−1.77717	0.00195	1.06607	0.00227	0.99959
b. HRS	−4.7162	0.00147	1.01045	0.00208	0.99941
